# The analysis of causal relationships between blood lipid levels and BMD

**DOI:** 10.1371/journal.pone.0212464

**Published:** 2019-02-22

**Authors:** Stacey S. Cherny, Maxim B. Freidin, Frances M. K. Williams, Gregory Livshits

**Affiliations:** 1 Department of Anatomy and Anthropology, Sackler Faculty of Medicine, Tel Aviv University, Tel Aviv, Israel; 2 Department of Epidemiology and Preventive Medicine, Sackler Faculty of Medicine, Tel Aviv University, Tel Aviv, Israel; 3 Department of Twin Research and Genetic Epidemiology, School of Life Course Science, King’s College London, London, United Kingdom; Institut Hospital del Mar d'Investigacions Mediques, SPAIN

## Abstract

Bone mineral density (BMD) and lipid levels are two of the most extensively studied risk factors for common diseases of aging, such as cardiovascular disease (CVD) and osteoporosis (OP). These two risk factors are also correlated with each other, but little is known about the molecular mechanisms behind this correlation. Recent studies revealed that circulating levels of several metabolites involved in the biosynthesis of androsterone correlate significantly with BMD and have the capacity to affect cholesterol and lipids levels. A main aim of the present study was to investigate the hypothesis that androsterone-related metabolites could provide a link between CVD and OP, as a common cause of lipid levels and BMD. The present study employed data from the NIHR BRC TwinsUK BioResource, comprising 1909 and 1994 monozygotic and dizygotic twin pairs, respectively, to address the causal relationships among BMD and lipids, and their associated metabolites, using reciprocal causation twin modelling, as well as Mendelian randomization (MR) using large publicly-available GWAS datasets on lipids and BMD, in conjunction with TwinsUK metabolite data. While results involving the twin modelling and MR analyses with metabolites were unable to establish a causal link between metabolite levels and either lipids or BMD, MR analyses of BMD and lipids suggest that lipid levels have a causal impact on BMD, which is consistent with findings from clinical trials of lipid-lowering drugs, which have also increased BMD.

## Introduction

Bone mineral density (BMD) and serum total cholesterol (TC) are among two of the most extensively studied clinically-oriented phenotypes, associated with the two of the most common polygenic age-related pathologies, osteoporosis (OP) and cardiovascular disease (CVD). There is an abundant literature showing correlation between these biomarkers and possible mechanisms underlying it [1–4], although the views and the data remain controversial [5,6].

In particular, it is unclear whether BMD and lipids are related due to pleiotropic genetic influences or through direct causal influence of one on the other. One of the most convincing studies employed conditional false-discovery rate and other approaches on large genome-wide association studies (GWAS) of BMD and cholesterol and found pleiotropy among the significant single nucleotide polymorphisms (SNPs) for the two phenotypes [4]. A comprehensive GWAS in a combined sample of the TwinsUK study and the Cooperative Health Research in the Region of Augsburg (KORA) study including nearly 8000 individuals identified 145 SNP associations with over 400 blood metabolites [7]. Of these, four independent SNPs were robustly associated with four metabolites previously shown to be involved in a single biological pathway and significantly correlated with BMD [8]: androsterone sulfate (ATS), dehydroisoandrosterone sulphate (DHEA-S), epiandrosterone sulfate (EAS), and 4-androsten-3beta,17beta-diol disulfate 2* (Δ^4^-dione). Two independent SNPs (rs7809615 on chromosome 7 [intronic variant in *TMEM225B*] and rs182420 on chromosome 19 [intronic variant in *LINC01595*]) were most associated with ATS and also had large and significant effects on DHEA-S and rs7809615 had a significant effect on EAS, Two additional independent SNPs were most associated with Δ^4^-dione (rs2762353 on chromosome 6 [intronic variant in *SLC17A1*] and rs4149056 on chromosome 12 [missense variant in *SLCO1B1*]) and these SNPs were not significantly associated with the other three metabolites. On the other hand, it is currently well established that cholesterol is involved in the biosynthesis of androsterone via steroidogenesis in the adrenal cortex [9,10]. These findings suggest possible mechanistic links between BMD and lipids via shared metabolic axis. However, studies evaluating possible casual associations between serum lipid profiles and BMD are few and contradictory.

In the current study, we addressed the question of whether the four steroid-pathway correlated metabolites causally influence BMD or the reverse, whether the four metabolites cause lipid levels or the reverse, and whether lipid levels cause BMD or the reverse. We applied two approaches: direction of causation twin modelling [11] and Mendelian randomization (MR) to investigate whether the metabolic pathways connecting BMD and lipid profiles could be causally identified. The twin modelling approach infers causation through differences in heritability and environmental variances of the variables of interest. The gold standard for establishing causation in medicine is the randomized controlled trial (RCT) [12], but performing RCTs is often not feasible or even ethical. However, with advances in genomics, MR was proposed and has been extensively implemented [13,14]. MR relies on the presence of genetic factors which cause a modifiable exposure and which do not directly cause the outcome of interest and only cause the outcome via modifying the exposure. Such genetic markers may then be used as instruments to examine whether there is in fact a causal link between the modifiable exposure (metabolite level, lipid level, or BMD) and the medically-relevant outcome (lipid level or BMD). Using large publicly-available datasets, combined with our metabolomic data, we addressed the same questions as in the twin causal modelling using MR.

## Materials and methods

### Subjects

The twin data used in the present study were from the TwinsUK Adult Twin Registry, described in detail previously [15]. The subsample employed in the present study comprised 1909 monozygotic (MZ) and 1994 dizygotic (DZ) twin pairs.

### Phenotypes

Metabolites were assayed from fasting plasma or serum samples by the service provider Metabolon, Inc. (Durham, NC, USA) [16]. The present study focusses on four of those metabolites: androsterone sulfate (ATS), dehydroisoandrosterone sulfate (DHEA-S), epiandrosterone sulfate (EAS), and 4-androsten-3beta, 17beta-diol disulfate 2* (Δ^4^-dione).

Lipid measures in the TwinsUK sample included four traits: total cholesterol (TC), high-density lipoprotein cholesterol (HDL-C), triglycerides (TG) and low-density lipoprotein cholesterol (LDL-C) calculated using the formula: LDL-C = ¾ (TC–HDL-C) [17]. Subjects were followed up and assessed at multiple time points, over a period of up to 17 years.

DEXA was used to measure BMD at the lumbar spine (L1 to L4) and hip regions (following manufacturer’s recommendations (QDR 4500W system, Hologic Inc, Bedford, MA) and described elsewhere [18]. In total, 15,491 DEXA scans were performed in 7056 twins during 17 years of follow-up; control scans were performed using the spine phantom.

### Data analysis

**Fig 1:**
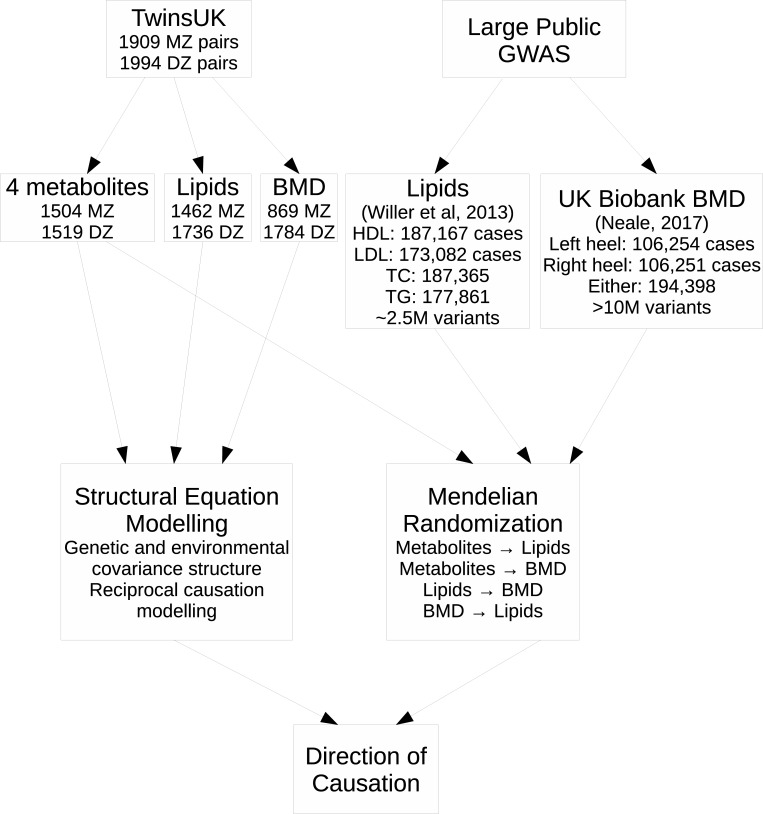
Flowchart of study design.

The overall analysis strategy, along with data employed, is presented in Fig 1.

All the analyses were carried out in the R statistical environment. DEXA scans and lipid profiles were obtained at multiple ages, therefore we used the lme4 package (https://cran.r-project.org/web/packages/lme4/lme4.pdf) to perform linear mixed-effects modelling to produce a single phenotype per individual by regressing out the age effect and using the intercept from those regressions as the individual score for each phenotype. This has the advantage of using all available data while adjusting for age.

**Fig 2:**
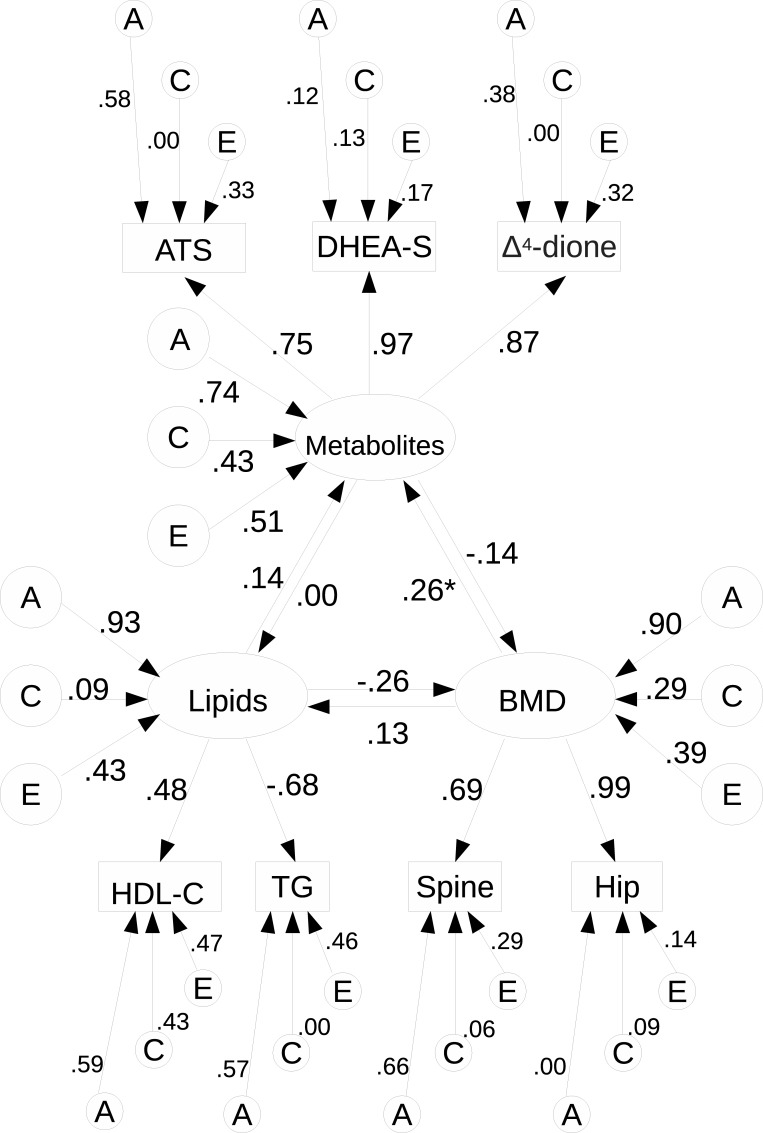
Path diagram of reciprocal causation twin model. Depiction is only for a single individual, including standardized parameter estimates from fitting the full model. For further explanations please see text.

Both standard univariate and multivariate twin modelling was performed using the umx package [19] to estimate the additive genetic (A) and shared in common by a twin pair (C) and nonshared (E) environmental variances and covariances. Direction of causation in twin modelling was performed using the OpenMX package [20–22]. We fitted the model illustrated in Fig 2, depicted for a single member of a twin pair. Models were fitted to the raw data and fit functions minimized with the CSOLNP optimizer [23]. The model contained three metabolites, two lipid measures, and two BMD measures (lumbar spine and left hip), with the omission of one metabolite and two other lipid measures as explained at the beginning of the results section. The model had a single latent factor for the metabolites, a latent factor for the lipids, and a latent factor for BMD. Parameters were fitted to allow for each latent factor to causally influence other latent factors in both possible directions (6 reciprocal causation parameters). The variances of each of the three latent factors were partitioned into genetic, shared environmental, and unique environmental components (9 parameters). To identify these three latent factors, the first loading on the observed variables for each factor was fixed to unity, leaving two free parameters loading on the metabolites, and one each on TG and left hip BMD (4 measurement model parameters). Finally, each observed variable had genetic, shared environmental, and non-shared environmental phenotype-specific variances estimated (3 components x 7 phenotypes = 21 parameters). All models equated means to be equal across twin pairs and zygosity (7 mean parameters). The total number of parameters estimated in the full model was therefore 47. Variables were re-scaled to have approximate unit variance to aid in optimization. Results of a standardized solution are presented, with all observed and latent variables standardized to unit variance.

Mendelian randomization analyses were performed using the MR-Base platform [24]. Four independent SNPs were chosen as instruments for the metabolites of interest. For ATS, the two independent SNPs which were most significantly associated in TwinsUK (rs7809615 on chromosome 7 and rs182420 on chromosome 19, significantly associated at p<10^−106^ and 2x10^-8^, respectively) were selected as instruments. These two SNPs also had large and significant effects on DHEA-S and rs7809615 had a significant effect on EAS, but no other independent SNPs were associated with these two metabolites, so no instruments were available for them. Additionally, the two independent SNPs most associated with Δ^4^-dione in TwinsUK (rs2762353 on chromosome 6 and rs4149056 on chromosome 12, significantly associated at p<3x10^-9^ and 2x10^-11^, respectively) were also selected as instruments. The large meta-GWAS of lipids [25] and GWAS for BMD established by heel ultrasound in the UK Biobank [26] were used as outcomes for this series of MR analyses (described below and in Fig 1). We also performed bidirectional MR with SNPs for lipids as instruments and BMD as outcome and the reverse. All MR analyses were performed using default options in MR-Base version 0.2.0 (17 December 2017), web app version 1.2.1 e646be (5 December 2018), and TwoSampleMR version 0.4.14, with clumping to prune SNPs in linkage disequilibrium.

## Results

### Twin modelling

Pearson correlations between phenotypes of interest (4 metabolites, 4 lipid measures, and 2 BMD measures) were estimated (Table 1). ATS and AES were highly correlated, as were LDL-C and TC. As expected, HDL-C was negatively correlated with the other lipid measures, but also with the BMD measures. TG was positively correlated with BMD. TC showed no correlation with BMD, while LDL-C was weakly correlated with one of the BMD measures.

**Table 1:** Phenotypic correlations.

	ATS	DHEA-S	AES	Δ^4^-dione	TC	HDL-C	TG	LDL-C	Spine	Hip
ATS	6031	6030	6001	4964	4956	4958	4898	4953	4125	4006
DHEA-S	0.72	6052	6006	4979	4971	4972	4913	4967	4142	4023
	*<* .0001									
EAS	0.94	0.74	6007	4944	4938	4940	4880	4935	4111	3992
	*<* .0001	*<* .0001								
Δ^4^-dione	0.64	0.84	0.65	4979	3949	3950	3892	3945	3301	3198
	*<* .0001	*<* .0001	*<* .0001							
TC	-0.02	-0.04	-0.03	-0.05	6371	6366	6302	6365	4471	4383
	*<* .02	*<* .002	*<* .05	*<* .004						
HDL-C	0.00	-0.01	0.00	-0.05	0.18	6374	6299	6365	4472	4384
	.7364	.3774	.7276	*<* .002	*<* .0001					
TG	-0.07	-0.10	-0.09	-0.04	0.31	-0.32	6304	6296	4402	4315
	*<* .0001	*<* .0001	*<* .0001	*<* .03	*<* .0001	*<* .0001				
LDL-C	-0.02	-0.04	-0.03	-0.03	0.93	-0.19	0.44	6365	4465	4377
	.1025	*<* .003	*<* .003	.0626	*<* .0001	*<* .0001	*<* .0001			
L Spine BMD	0.04	0.05	0.04	0.07	-0.02	-0.06	0.06	0.01	5320	5161
	*<*0.01	*<* .002	*<* .02	*<* .0001	0.1838	*<* .0001	*<* .0004	.6921		
Left Hip BMD	0.07	0.10	0.08	0.14	-0.01	-0.12	0.07	0.04	0.68	5171
	*<* .0001	*<* .0001	*<* .0001	*<* .0001	0.6164	*<* .0001	*<* .0001	*<* .02	*<* .0001	

Correlations are presented in the lower triangle, with *p*-values underneath them, and sample sizes in the upper triangle.

All metabolites were significantly correlated with TG and TC, while only Δ^4^-dione was correlated with HDL-C and only DHEA-S and EAS were correlated with LDL-C. To avoid collinearity due to strong correlation of ATS with EAS (r = 0.94) and LDL-C with TC (r = 0.93), we omitted EAS and LDL-C from the standard multivariate genetic model (8 phenotypes analyzed). Also, we used only TG and HDL-C for the reciprocal causation modelling (7 phenotypes analyzed).

A multivariate (multiphenotype) full genetic model was first fitted to the twin data to explore the underlying genetic and environmental architecture. For estimating the genetic and environmental covariance matrices among the 8 measures, 36 genetic, 36 shared environmental, and 36 nonshared environmental variances and covariances were directly estimated (108 parameters). In addition, means were estimated for each variable, equated across twin pairs and zygosity (8 parameters). Tables 2–4 present the genetic, shared environmental, and nonshared environmental phenotypically-standardized covariance matrices, respectively. Summing these three covariance matrices yields the expected phenotypic correlation matrix among the 8 measures. All phenotypes were highly heritable, ranging from .50 for TC to .82 for lumbar spine BMD (diagonal elements in Table 2), and all the heritability estimates were statistically significant (*p* < .001). Shared environmental influences were generally low, ranging from -.01 for triglycerides to .21 for DHEA-S (diagonal elements in Table 3). While negative variances make no theoretical sense, in the standard twin model, they do imply that the DZ correlation is less than half the MZ correlation, which would suggest dominance variance rather than shared environmental variance, but this negative parameter was not statistically significant. Finally, nonshared environmental variance (diagonal elements in Table 4), while small, is somewhat higher for the metabolite measures and lipids, on average, than for BMD.

**Table 2:** Phenotypically standardized genetic variance components.

	ATS	DHEA-S	Δ^4^-dione	TC	HDL-C	TG	Spine BMD	Hip BMD
ATS	0.68***							
DHEA-S	0.41***	0.53***						
Δ^4^-dione	0.37***	0.47***	0.59***					
TC	0.01	0.02	0.02	0.50***				
HDL-C	0.07*	0.05	−0.01	0.05	0.55***			
TG	−0.01	−0.02	0.04	0.15***	−0.27***	0.72***		
L Spine BMD	0.07*	0.05	0.07*	−0.06	−0.09**	0.05	0.82***	
Left Hip BMD	0.08*	0.08**	0.10**	−0.01	−0.10**	0.06**	0.54***	0.75***

***p < .001

**p < .01

*p < .05

**Table 3:** Phenotypically standardized shared environmental variance components.

	ATS	DHEA-S	Δ^4^-dione	TC	HDL-C	TG	Spine BMD	Hip BMD
ATS	0.07							
DHEA-S	0.13***	0.21***						
Δ^4^-dione	0.09*	0.15***	0.09**					
TC	−0.04	−0.07*	−0.07*	0.17***				
HDL-C	−0.07*	−0.05	−0.02	0.07*	0.19***			
TG	−0.05	−0.06	−0.07*	0.07*	0.00	−0.01		
L Spine BMD	−0.02	0.01	0.01	0.04	0.03	−0.01	0.02	
Left Hip BMD	0.00	0.02	0.03	−0.01	−0.01	−0.01	0.04	0.08*

***p < .001

**p < .01

*p < .05

**Table 4:** Phenotypically standardized non-shared environmental variance components.

	ATS	DHEA-S	Δ^4^-dione	TC	HDL-C	TG	Spine BMD	Hip BMD
ATS	0.25#							
DHEA-S	0.18***	0.26#						
Δ^4^-dione	0.18*	0.22***	0.31#					
TC	0.01	0.01	0.01	0.33#				
HDL-C	−0.00	−0.01	−0.01	0.07***	0.26#			
TG	−0.02	−0.02*	−0.01	0.10***	−0.06***	0.29#		
L Spine BMD	−0.00	−0.00	−0.00	0.01	0.00	0.02**	0.16#	
Left Hip BMD	0.00	0.00	0.01	0.01	−0.01	0.02*	0.10***	0.17#

***p < .001

**p < .01

*p < .05

#not tested, since these include measurement error

Examining the sources of variance underlying the correlations among measures, first genetic variation within domains, all metabolites were significantly genetically inter-correlated (off-diagonal elements in Table 2). All but one (TC with HDL-C) genetic covariance among lipids was significant, and the genetic covariance between BMD measures was substantial and significant. Examining the genetic components of cross-domain correlation, of all the genetic covariances between metabolites and lipids, only ATS with HDL-C was significant, though modest (.07). Five of 6 genetic covariances between metabolites and BMD measures were significant. Finally, of the lipids, only HDL-C was significantly genetically correlated with both BMD measures, while TG was correlated with hip BMD.

Shared environment also contributed significantly to all correlations between the metabolites (off-diagonal elements in Table 3), but did not significantly contribute to BMD variance or covariance, nor to lipid covariances. Some of the covariation between metabolites and lipids was due to shared environmental influences, with 4 out of 9 covariances statistically significant, although all rather small. Finally, the shared environment did not contribute to the correlations between metabolites and BMD measures.

Nonshared environment contributes substantially to the covariances between metabolites, likely reflecting day-to-day co-variation of metabolites involved in the same biochemical pathway (off-diagonal elements in Table 4). Nonshared environment also contributed significantly to the covariances among lipids and among BMD measures, though the magnitude of the covariances among lipids were smaller than for BMD. Nonshared environment generally did not contribute to correlations between metabolites and lipids, between metabolites and BMD, nor between lipids and BMD, though a few of the covariances were statistically significant.

Next, we fitted a direction of causation twin model to the data, with common factors underlying the covariation among metabolites, lipids, and BMD measures, with standardized parameter estimates from the full model presented in Fig 2. We tested all parameters (with the exception of measure-specific E, which contains measurement error) against the full model (Table 5). We first tested the common factor A, C, and E variances. All three common factors (metabolites, lipids, and BMD) had significant genetic and nonshared environmental variance components, but only the metabolites had a significant shared family environmental component. Looking at the measure-specific variances, DHEA-S, TG, and hip BMD did not have specific genetic variance components (the genetic common factor was sufficient to explain the genetic variance present), but the other four measures did. Only HDL-C had a significant measure-specific shared environmental component.

**Table 5:** Model comparisons.

Test		ep^1^	-2LL	df	diff LL	diff df	p
Full model		47	91762.47	40085			
drop A	Metabolites	46	91836.26	40086	73.78595	1	8.70642e-18
	Lipids	46	91836.26	40086	73.78595	1	8.706414e-18
	BMD	46	91920.44	40086	157.9722	1	3.138427e-36
drop C	Metabolites	46	91777.43	40086	14.95758	1	0.0001099557
	Lipids	46	91762.48	40086	0.01065589	1	0.9177825
	BMD	46	91765.26	40086	2.783978	1	0.09521143
drop E	Metabolites	46	91850.62	40086	88.14331	1	6.08798e-21
	Lipids	46	91833.07	40086	70.59596	1	4.384147e-17
	BMD	46	91844.32	40086	81.84881	1	1.469053e-19
drop A	ATS	46	92048.62	40086	286.1465	1	3.437298e-64
	DHEA-S	46	91763.41	40086	0.9388292	1	0.3325791
	Δ^4^-dione	46	91834.34	40086	71.87202	1	2.296169e-17
	HDL-C	46	91773.70	40086	11.22382	1	0.0008075405
	TG	46	91765.99	40086	3.515016	1	0.06081508
	Spine BMD	46	91806.07	40086	43.60174	1	4.024813e-11
	Hip BMD	46	91762.47	40086	0	1	1
drop C	ATS	46	91762.47	40086	0	1	1
	DHEA-S	46	91764.67	40086	2.194953	1	0.1384635
	Δ^4^-dione	46	91762.47	40086	0	1	1
	HDL-C	46	91784.08	40086	21.60901	1	3.342781e-06
	TG	46	91762.47	40086	0	1	1
	Spine BMD	46	91762.50	40086	0.0267201	1	0.870154
	Hip BMD	46	91762.55	40086	0.07633446	1	0.7823277
drop Metab	→Lipids	46	91762.47	40086	0.0002280452	1	0.9879515
	←Lipids	46	91763.49	40086	1.016024	1	0.313464
	⇔Lipids	45	91765.74	40087	3.265054	2	0.1954351
drop Metab	→BMD	46	91765.63	40086	3.159034	1	0.07550787
	←BMD	46	91772.22	40086	9.749522	1	0.001793694
	⇔BMD	45	91773.54	40087	11.06272	2	0.003960596
drop Lipids	→BMD	46	91763.15	40086	0.6783092	1	0.4101696
	←BMD	46	91762.65	40086	0.1791145	1	0.6721353
	⇔BMD	45	91768.11	40087	5.642096	2	0.0595435

^1^number of estimated parameters in model

To test the direction of causation, each causal path was dropped and tested against the full model that allows for reciprocal causation. No evidence for causal relationships was found after Bonferroni correction (p = 0.05/6 = .008 for 6 possible causal paths) except for the causal path from BMD to the metabolite common factor. Looking at the underlying correlations among latent factors, ultimately they were likely too low to establish direction of causation, though not too low to establish correlation. As can be seen when testing both causal directions simultaneously, the BMD common factor was found to be phenotypically correlated with just the metabolites, with the other two tests being not statistically significant.

### Mendelian randomization

We used Mendelian randomization to test the hypothesis that changes in the levels of ATS and Δ^4^-dione would cause changes in the lipid and BMD levels (Table 6). We present single SNP tests as well as inverse variance weighted tests of the two SNPs together. Reverse causation couldn't be explored due to small sample size of the metabolite dataset.

**Table 6:** MR tests for metabolite instruments on lipid and BMD outcomes.

Exposure	Outcome	SNP	*b*	se	*p*
ATS	HDL	rs7809615	-0.0379	0.0272	0.164
		rs182420	0.0588	0.0784	0.453
		Both (IVW)	-0.0275	0.0300	0.359
	LDL	rs7809615	0.0193	0.0296	0.515
		rs182420	0.1510	0.0843	0.073
		Both (IVW)	0.0337	0.0411	0.413
	TC	rs7809615	0.0279	0.0289	0.334
		rs182420	0.2549	0.0824	0.002
		Both (IVW)	0.0528	0.0709	0.457
	Triglycerides	rs7809615	0.0282	0.0269	0.294
		rs182420	0.2980	0.0765	9.7×10^*−*5^
		Both (IVW)	0.0580	0.0845	0.492
	BMD left	rs7809615	0.0389	0.0244	0.111
		rs182420	-0.0658	0.0965	0.496
		Both (IVW)	0.0326	0.0249	0.191
	BMD right	rs7809615	0.0530	0.0243	0.029
		rs182420	-0.2002	0.0959	0.037
		Both (IVW)	0.0377	0.0602	0.531
	BMD total	rs7809615	0.0540	0.0183	0.003
		rs182420	-0.1625	0.0716	0.023
		Both (IVW)	0.0407	0.0520	0.433
Δ^4^-dione	HDL	rs2762353	0.1094	0.1062	0.303
		rs4149056	0.0060	0.0920	0.948
		Both (IVW)	0.0503	0.0696	0.470
	LDL	rs2762353	0.1375	0.1156	0.234
		rs4149056	-0.1520	0.1000	0.129
		Both (IVW)	-0.0281	0.1432	0.844
	TC	rs2762353	0.2125	0.1094	0.052
		rs4149056	-0.0380	0.0960	0.692
		Both (IVW)	0.0710	0.1242	0.568
	Triglycerides	rs2762353	0.0719	0.1031	0.486
		rs4149056	0.4800	0.0940	3.3×10^*−*7^
		Both (IVW)	0.2948	0.2032	0.147
	BMD left	rs2762353	0.1369	0.1311	0.296
		rs4149056	0.2249	0.1166	0.054
		Both (IVW)	0.1860	0.0871	0.033
	BMD right	rs2762353	0.0984	0.1302	0.450
		rs4149056	0.2820	0.1159	0.015
		Both (IVW)	0.2008	0.0912	0.028
	BMD total	rs2762353	0.0219	0.0981	0.824
		rs4149056	0.2014	0.0869	0.020
		Both (IVW)	0.1225	0.0891	0.169

We employed a conservative Bonferroni multiple testing correction for 42 tests (2 metabolites x 3 instruments (two SNPs and joint test) x 7 traits) giving a *p*-value threshold of < .0012. We found evidence that ATS levels causally influence TG levels for one of the SNP instruments only (p < 0.0001). There is also suggestive evidence for ATS levels influencing TC and right and total BMD from one SNP instrument only. There is also suggestive evidence that Δ^4^-dione levels influence right and left heel BMD, though statistical significance was not attained. The rs4149056 SNP, however, had a significant effect on TG levels, but not on HDL-C, LDL-C, or TC.

We also explored the question of whether lipid levels cause BMD, BMD causes lipid levels, or both (Table 7). We used lipid level and BMD SNPs from the same public datasets as instruments. We calculated several MR test statistics, though present results for the new weighted mode estimator, which has been shown to be robust even when the majority of instruments violate assumptions of MR [27].

**Table 7:** Bidirectional MR tests for lipids and BMD.

			Weighted mode			MR-Egger Intercept		
Outcome	Exposure	*n* SNPs	*b*	se	*p*	*a*	se	*p*
Heel BMD left	HDL cholesterol	87	0.01399	0.01842	0.4495	-0.0064	0.0023	0.00718
Heel BMD right			0.001238	0.01705	0.9423	-0.0056	0.0023	0.0187
Heel BMD total			-0.005529	0.01254	0.6602	-0.0045	0.0022	0.0472
Heel BMD left	LDL cholesterol	78	-0.02709	0.01077	0.01396	-0.0023	0.0015	0.128
Heel BMD right			-0.02755	0.01183	0.02244	-0.0031	0.0016	0.0569
Heel BMD total			-0.03555	0.00947	0.0003356	-0.0015	0.0015	0.34
Heel BMD left	Total cholesterol	86	-0.0195	0.01455	0.1836	-0.0024	0.0016	0.125
Heel BMD right			-0.01757	0.01372	0.2037	-0.0024	0.0016	0.136
Heel BMD total			-0.02885	0.01066	0.008255	-0.0019	0.0015	0.198
Heel BMD left	Triglycerides	54	-0.04249	0.02048	0.04285	0.0025	0.0029	0.39
Heel BMD right			-0.01824	0.01992	0.3638	0.0017	0.0029	0.573
Heel BMD total			-0.001395	0.01542	0.9282	0.0023	0.0027	0.407
HDL cholesterol	Heel BMD left	94	-0.01867	0.03411	0.5854	-0.00068	0.0018	0.713
LDL cholesterol			-0.06013	0.02546	0.0203	0.0012	0.0017	0.502
Total cholesterol			-0.05498	0.02243	0.01612	-6e-04	0.0016	0.711
Triglycerides			-0.03376	0.02491	0.1786	-0.0011	0.0015	0.479
HDL cholesterol	Heel BMD right	80	-0.04655	0.03048	0.1308	0.00058	0.0021	0.784
LDL cholesterol			-0.04991	0.02869	0.08576	0.0015	0.002	0.434
Total cholesterol			-0.04585	0.02786	0.1038	8e-04	0.0019	0.671
Triglycerides			-0.01676	0.02914	0.5668	4e-05	0.0017	0.982
HDL cholesterol	Heel BMD total	157	0.02457	0.03074	0.4254	0.00049	0.0015	0.739
LDL cholesterol			0.0001458	0.03974	0.9971	-0.00036	0.0014	0.797
Total cholesterol			-0.002038	0.02785	0.9418	-0.00034	0.0014	0.808
Triglycerides			-0.05696	0.02938	0.05432	-0.0012	0.0013	0.34

There is evidence of LDL-C levels causing BMD. Even with a conservative Bonferroni correction for 8 tests, 4 lipid levels causing total BMD and total BMD causing 4 lipid measures (threshold of .05/8 = .0063), LDL-C significantly causes total heel BMD, with *p*-values also nominally significant for right and left heel measures separately. There was no evidence of directional horizontal pleiotropy for LDL-C, with the MR-Egger intercepts found not to be statistically significant. In addition, with the exception of HDL exposure on BMD, all other MR-Egger intercepts were also not nominally statistically significant. All tests of trait heterogeneity, however, were statistically significant.

**Fig 3:**
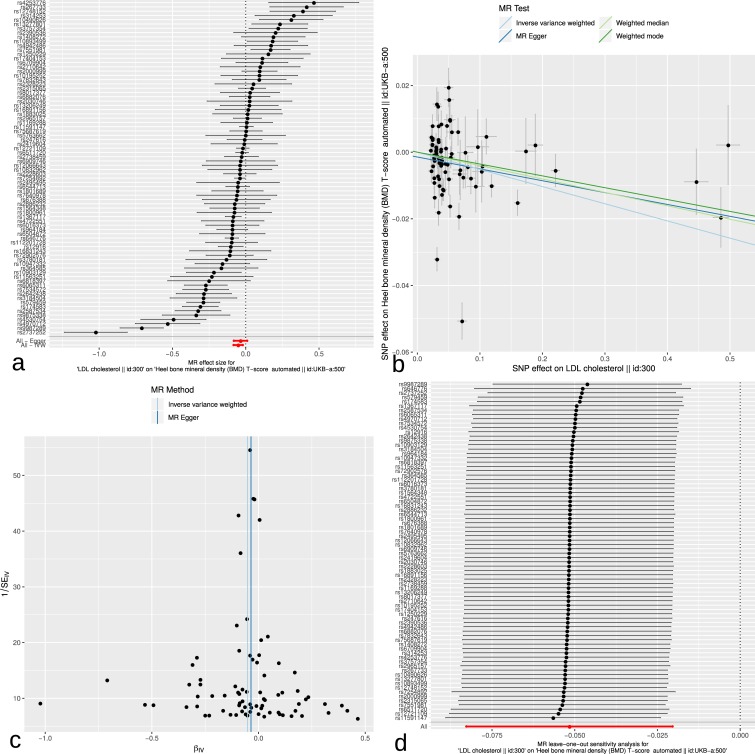
Plots of the MR analysis of the LDL-C exposure predicting total heel BMD outcome. Panel a: forest plot of individual SNP effects, along with 95% confidence intervals. MR-Egger and IVW results are also presented. Panel b: a comparison of the slopes obtained from the various MR methods employed. Panel c: funnel plot showing causal effect of each SNP against the inverse of the standard error of the estimate. The vertical lines show the MR estimates using all SNPs for MR-Egger and IVW methods. Panel d: leave-one-out sensitivity analysis, using the IVW method, along with IVW overall result in red.

Fig 3 contains plots of the MR analysis of the LDL-C exposure on total heel BMD outcome. Panel a presents the forest plot of individual SNP effects, along with 95% confidence intervals. Its symmetry suggests no pleiotropic effects. While the MR-Egger regression wasn’t statistically significant, the inverse variance weighted (IVW) analysis did yield an overall significant effect of LDL-C. Panel b compares the slopes from the various MR methods employed and shows the weighted mode estimator that we presented above has a slope very similar to the other methods employed. Panel c presents the funnel plot showing the relationship between the causal effect of LDL-C on BMD estimated by each SNP against the inverse of the standard error of the estimate. The vertical lines show the MR estimates using all SNPs for MR-Egger and IVW methods. The relative symmetry of the funnel plot suggests a lower risk of horizontal pleiotropy leading to unreliable associations, as does the MR-Egger intercept. Finally, panel d presents the results of a leave-one-out sensitivity analysis, using the IVW method, and suggests no major outliers were present.

## Discussion

There are many studies on subjects of different ethnic backgrounds that have demonstrated a significant association between serum lipid profile, specifically TC and LDL-C, and BMD, both in the general population and in alcoholic people [28]. These observations raise the question of possible underlying mechanisms that explain the association between serum lipids and bone metabolism. One such mechanism could be related to metabolism of endogenous steroid hormones. TC is a major structural component of membranes and a substrate for the biosynthesis of other steroids, including ATS and DHEA-S [9]. On the other hand, the important role of endogenous and exogenous steroid hormones in osteoporosis etiology is well known. Recently, a significant correlation of the metabolites selected in this study with hip and spine BMD was reported [8]. The major aim of the present study was therefore twofold: 1) to test the hypothesis that three physiological facets (lipids, BMD, and steroid hormones-related metabolites) have a common genetic background, and 2) to examine possible causal relationships. Using MR, we found some evidence of ATS levels influencing TG and TC, as well as BMD. Also, it appears Δ^4^-dione may be causing BMD and TG levels. Given lack of statistical significance for most of these association, or significance for one SNP instrument and not the other, the causal relationship between these metabolites and lipids and BMD has not been convincingly demonstrated. In addition, our results are not completely consistent with a recent analysis of TwinsUK data, which found evidence for both these metabolite levels influencing BMD, and this result was also replicated in a Hong Kong sample [8]. However, our approach used BMD outcome measures from the UK Biobank, rather than from the same individuals on whom metabolites were assayed. Furthermore, with only two SNP instruments available for each of the metabolites of interest, the study is not optimally powered nor can we check for violations of MR assumptions, as can be done when employing multiple instruments.

A number of studies looked at the correlation between serum HDL and BMD [29], but no consistent relationship was found, with some studies finding positive correlations and others negative correlations. Addressing issues of causation, one study found lower TC and LDL levels, along with an expected higher BMD in postmenopausal women taking hormone replacement therapy (HRT) compared to those who were not [30]. This suggests either BMD levels influence cholesterol levels or that there is a common cause of both BMD and cholesterol, such as estrogen levels. Our MR results showed no evidence of BMD causing lipid levels. In a recent meta-analysis, statin use was linked to increased BMD, though not to reduced fracture [31], suggesting a causal relationship from cholesterol to BMD, or a common cause of the two. Consistent with this, our MR results demonstrated evidence of LDL-C levels influencing BMD.

Direction of causation modelling in twin studies has been implemented successfully in a number of medical and behavioral phenotypes [11,32–36]. While the approach relies on very different data and methods, it can address the same questions of causation as RCT or MR studies. The major limiting factor in the twin study approach to causation is that it requires the traits of interest to differ in heritability and shared environment, otherwise the approach lacks power. Our twin modeling demonstrated significant genetic overlap among the analyzed traits, but reciprocal causation modeling of twin data did not yield definitive results. This is likely explained by the little difference in the heritability estimates between the most heritable traits (BMD) and the least heritable traits (metabolites). Shared environmental variance was also minimal in all the measures examined, further limiting the power of our study design. A further limitation of the twin modelling is that because TwinsUK is a community-based sample, detailed clinically-relevant information is unavailable, including such important covariates as use of lipid-lowering or BMD-enhancing drugs, or use of environmental interventions, potentially further attenuating power.

The animal model literature suggests shared genes predispose to both HDL-C and BMD [29]. Our twin analyses also found a significant genetic correlation between HDL-C and BMD. However, using LD-score regression and results from the same large public datasets that we analyzed [37], no significant genetic correlation between HDL levels and BMD levels was shown, while the genetic correlation was -.14 for LDL and lumbar spine BMD (p = .0376) and -.145 for LDL with neck BMD (p = .0109). There was also a genetic correlation of -.137 for TC and lumbar spine BMD (p = .0135) and -.138 for TC with neck BMD (p = .0028), but no statistically significant genetic correlation for TG levels. Our twin analyses failed to find a significant genetic correlation between TC (highly correlated with LDL-C) and BMD, however.

In summary, our study confirmed previous findings on the relationship between lipid levels and BMD, as well as their correlation with the metabolites we examined. The twin modelling failed to establish the direction of causation. However, the MR analyses suggest that causation goes from LDL-C level to BMD, consistent with previous studies. Further studies, particularly of the metabolic factors, would benefit from a larger sample of twins both for reciprocal causation modelling and also to test BMD and lipid genetic variants effects on metabolite levels.

## References

[1] Go J-H, Song Y-M, Park J-H, Park J-Y, Choi Y-H. Association between Serum Cholesterol Level and Bone Mineral Density at Lumbar Spine and Femur Neck in Postmenopausal Korean Women. Korean J Fam Med. 2012;33: 166–173. 10.4082/kjfm.2012.33.3.166 22787539 PMC3391642

[2] Jeong T-D, Lee W, Choi S-E, Kim JS, Kim H-K, Bae SJ, et al Relationship between serum total cholesterol level and serum biochemical bone turnover markers in healthy pre- and postmenopausal women. BioMed Res Int. 2014;2014: 398397 10.1155/2014/398397 24949440 PMC4052088

[3] Jiang X-Y, Chen Y, Xu L, Li X, Cao F-F, Li L, et al Association of LPR5 polymorphism with bone mass density and cholesterol level in population of Chinese Han. Exp Clin Endocrinol Diabetes Off J Ger Soc Endocrinol Ger Diabetes Assoc. 2010;118: 388–391. 10.1055/s-0029-1225613 20146170

[4] Lin X, Peng C, Greenbaum J, Li Z-F, Wu K-H, Ao Z-X, et al Identifying potentially common genes between dyslipidemia and osteoporosis using novel analytical approaches. Mol Genet Genomics MGG. 2018;293: 711–723. 10.1007/s00438-017-1414-1 29327327 PMC5949092

[5] Ghadiri-Anari A, Mortezaii-Shoroki Z, Modarresi M, Dehghan A. Association of lipid profile with bone mineral density in postmenopausal women in Yazd province. Int J Reprod Biomed Yazd Iran. 2016;14: 597–602. PMC5054297 27738662

[6] Tankó LB, Christiansen C, Cox DA, Geiger MJ, McNabb MA, Cummings SR. Relationship between osteoporosis and cardiovascular disease in postmenopausal women. J Bone Miner Res Off J Am Soc Bone Miner Res. 2005;20: 1912–1920. 10.1359/JBMR.050711 16234963

[7] Shin SY, Fauman EB, Petersen AK, Krumsiek J, Santos R, Huang J, et al An atlas of genetic influences on human blood metabolites. Nat Genet. 2014;46: 543–550. 10.1038/ng.2982 24816252 PMC4064254

[8] Moayyeri A, Cheung C-L, Tan KC, Morris JA, Cerani A, Mohney RP, et al Metabolomic Pathways to Osteoporosis in Middle-Aged Women: A Genome-Metabolome-Wide Mendelian Randomization Study. J Bone Miner Res Off J Am Soc Bone Miner Res. 2018;33: 643–650. 10.1002/jbmr.3358 29232479 PMC5972819

[9] Wang DQ-H, Cohen DE. CHAPTER 3—Absorption and Excretion of Cholesterol and Other Sterols In: Ballantyne CM, editor. Clinical Lipidology. Philadelphia: W.B. Saunders; 2009 pp. 26–44. 10.1016/B978-141605469-6.50007-X

[10] Rosol TJ, Yarrington JT, Latendresse J, Capen CC. Adrenal gland: structure, function, and mechanisms of toxicity. Toxicol Pathol. 2001;29: 41–48. 10.1080/019262301301418847 11215683

[11] Heath AC, Kessler RC, Neale MC, Hewitt JK, Eaves LJ, Kendler KS. Testing hypotheses about direction of causation using cross-sectional family data. Behav Genet. 1993;23: 29–50. 8476389 10.1007/BF01067552

[12] Cartwright N. What are randomised controlled trials good for? Philos Stud. 2010;147: 59 10.1007/s11098-009-9450-2

[13] Smith GD, Ebrahim S. “Mendelian randomization”: can genetic epidemiology contribute to understanding environmental determinants of disease? Int J Epidemiol. 2003;32: 1–22. 12689998 10.1093/ije/dyg070

[14] Davey Smith G, Hemani G. Mendelian randomization: genetic anchors for causal inference in epidemiological studies. Hum Mol Genet. 2014;23: R89–98. 10.1093/hmg/ddu328 25064373 PMC4170722

[15] Moayyeri A, Hammond CJ, Hart DJ, Spector TD. The UK Adult Twin Registry (TwinsUK Resource). Twin Res Hum Genet. 2013;16: 144–149. 10.1017/thg.2012.89 23088889 PMC3927054

[16] Illig T, Gieger C, Zhai G, Romisch-Margl W, Wang-Sattler R, Prehn C, et al A genome-wide perspective of genetic variation in human metabolism. Nat Genet. 2010;42: 137–141. 10.1038/ng.507 20037589 PMC3773904

[17] de Cordova CMM, de Cordova MM. A new accurate, simple formula for LDL-cholesterol estimation based on directly measured blood lipids from a large cohort. Ann Clin Biochem. 2013;50: 13–19. 10.1258/acb.2012.011259 23108766

[18] Arden NK, Spector TD. Genetic influences on muscle strength, lean body mass, and bone mineral density: a twin study. J Bone Miner Res Off J Am Soc Bone Miner Res. 1997;12: 2076–2081. 10.1359/jbmr.1997.12.12.2076 9421240

[19] Bates TC, Maes HH, Neale MC. umx: Twin and Path-based Structural Equation Modeling in R [Internet]. PeerJ Inc.; 2017 Oct. Report No.: e3354v1. 10.7287/peerj.preprints.3354v1 30944056

[20] Hunter MD. State Space Modeling in an Open Source, Modular, Structural Equation Modeling Environment. Struct Equ Model Multidiscip J. 2018;25: 307–324. 10.1080/10705511.2017.1369354

[21] Neale MC, Hunter MD, Pritikin JN, Zahery M, Brick TR, Kirkpatrick RM, et al OpenMx 2.0: Extended Structural Equation and Statistical Modeling. Psychometrika. 2016;81: 535–549. 10.1007/s11336-014-9435-8 25622929 PMC4516707

[22] Pritikin JN, Hunter MD, Boker SM. Modular Open-Source Software for Item Factor Analysis. Educ Psychol Meas. 2015;75: 458–474. 10.1177/0013164414554615 27065479 PMC4822086

[23] Zahery M, Maes HH, Neale MC. CSOLNP: Numerical Optimization Engine for Solving Non-linearly Constrained Problems. Twin Res Hum Genet. 2017;20: 290–297. 10.1017/thg.2017.28 28535831 PMC5750059

[24] Hemani G, Zheng J, Wade K, Laurin C, Elsworth B, Burgess S, et al MR-Base: a platform for systematic causal inference across the phenome using billions of genetic associations. bioRχiv. 2016; 10.1101/078972

[25] Willer CJ, Schmidt EM, Sengupta S, Peloso GM, Gustafsson S, Kanoni S, et al Discovery and refinement of loci associated with lipid levels. Nat Genet. 2013;45: 1274–1283. 10.1038/ng.2797 24097068 PMC3838666

[26] Neale B. Details and considerations of the UK Biobank GWAS [Internet]. 2017. Available: http://www.nealelab.is/blog/2017/9/11/details-and-considerations-of-the-uk-biobank-gwas

[27] Hartwig FP, Davey Smith G, Bowden J. Robust inference in two-sample Mendelian randomisation via the zero modal pleiotropy assumption. bioRχiv. 2017; 10.1101/126102 PMC5837715 29040600

[28] Martín-González C, González-Reimers E, Quintero-Platt G, Cabrera-García P, Romero-Acevedo L, Gómez-Rodríguez MÁ, et al Lipid profile and bone mineral density in heavy alcoholics. Clin Nutr Edinb Scotl. 2017; 10.1016/j.clnu.2017.10.008 29089152

[29] Ackert-Bicknell CL. HDL cholesterol and bone mineral density: is there a genetic link? Bone. 2012;50: 525–533. 10.1016/j.bone.2011.07.002 21810493 PMC3236254

[30] Makovey J, Chen JS, Hayward C, Williams FM, Sambrook PN. Association between serum cholesterol and bone mineral density. Bone. 2009;44: 208–213. 10.1016/j.bone.2008.09.020 18983946

[31] Wang Z, Li Y, Zhou F, Piao Z, Hao J. Effects of Statins on Bone Mineral Density and Fracture Risk: A PRISMA-compliant Systematic Review and Meta-Analysis. Med Baltim. 2016;95: e3042. 10.1097/MD.0000000000003042 PMC4900696 27258488

[32] Duffy DL, Martin NG. Inferring the direction of causation in cross-sectional twin data: theoretical and empirical considerations. Genet Epidemiol. 1994;11: 483–502. 10.1002/gepi.1370110606 7713391

[33] Gillespie NA, Gehrman P, Byrne EM, Kendler KS, Heath AC, Martin NG. Modeling the direction of causation between cross-sectional measures of disrupted sleep, anxiety and depression in a sample of male and female Australian twins. J Sleep Res. 2012;21: 675–683. 10.1111/j.1365-2869.2012.01026.x 22738694 PMC3461239

[34] Gillespie NA, Zhu G, Neale MC, Heath AC, Martin NG. Direction of causation modeling between cross-sectional measures of parenting and psychological distress in female twins. Behav Genet. 2003;33: 383–396. 14574138 10.1023/a:1025365325016

[35] Toulopoulou T, van Haren N, Zhang X, Sham PC, Cherny SS, Campbell DD, et al Reciprocal causation models of cognitive vs volumetric cerebral intermediate phenotypes for schizophrenia in a pan-European twin cohort. Mol Psychiatry. 2015;20: 1386–1396. 10.1038/mp.2014.152 25450228

[36] Verhulst B, Estabrook R. Using genetic information to test causal relationships in cross-sectional data. J Theor Polit. 2012;24: 328–344. 10.1177/0951629812439348 23946557 PMC3740451

[37] Zheng J, Erzurumluoglu AM, Elsworth BL, Kemp JP, Howe L, Haycock PC, et al LD Hub: a centralized database and web interface to perform LD score regression that maximizes the potential of summary level GWAS data for SNP heritability and genetic correlation analysis. Bioinformatics. 2017;33: 272–279. 10.1093/bioinformatics/btw613 27663502 PMC5542030

